# Supplementary material for: Predicting post-operative right ventricular failure using video-based deep learning
Source: Nat Commun. 2021 Aug 31;12:5192. doi: 10.1038/s41467-021-25503-9 (PMC8408163; doi:10.1038/s41467-021-25503-9)
Supplement: Supplementary file 1 — Supplementary Information [file 41467_2021_25503_MOESM1_ESM.pdf]

Supplementary Information

Right Ventricular Failure definitions:<br>Right Atrial Pressure > 16mm Hg.<br>**AND**<br>Hepatic (total bilirubin > 2.0 mg/dl) or renal dysfunction (creatinine > 2.0 mg/dl)

Index Admission MCS ARC Definitions

**RV Failure**	Meets both criteria for RV Failure<br>**AND**<br>Prolonged post-implant inotropes, inhaled nitric oxide or intravenous vasodilators continued beyond post-op day 14 following LVAD implant<br>**OR**<br>Need for right ventricular assist device at any time following LVAD implant<br>**OR**<br>Death during the LVAD implants hospitalization with RHF as the primary cause.

Supplementary Table 1: MCS ARC definitions of RV failure. Updated RV failure definitions and index admission criteria for RV failure as defined by the Mechanical Circulatory Support Academic Research Consortium as of August 2020.

Scoring System	Pre-operative parameters
**CRITT Score**	Right atrial pressure (RAP) > 15<br>Severe RV dysfunction (Qualitative echocardiography report)<br>Severe Tricuspid Regurgitation<br>Tachycardia (HR > 100)<br>Ventilator requirement
**Penn Score**	Cardiac Index (CI) ≤ 2.2<br>RV stroke work index (RVSWI) ≤ 0.25<br>Creatinine ≥ 1.9 mg/dl<br>History of previous cardiac surgery<br>Severe RV dysfunction (Qualitative echocardiography report)<br>Systolic blood pressure (SBp) ≤ 96 mm Hg

Supplementary Table 2: Scoring parameters for CRITT and Penn Score. For each criterion listed, a score of '1' is assigned and the CRITT score is calculated as the sum of all components. For example, a patient with RAP > 15, tachycardia, and severe tricuspid regurgitation would have a CRITT score of 3. The Penn score is calculated by similarly assigning a score of '1' for each criterion listed, the calculation however is (18 x CI) + (18 x RVSWI) + (17 x Creatinine) + (16 x Previous cardiac surgery) + (16 x Severe RV dysfunction) + (13 x SBp)

	Validation (N=135)	Training (N=467)	Testing (N=121)
**RV Failure Status**			
**No**	105 (77.8%)	345 (73.9%)	91 (75.2%)
**Yes**	30 (22.2%)	122 (26.1%)	30 (24.8%)
**Age (years)**			
Mean (sd)	57.72 (13.34)	56.86 (13.28)	59.47 (12.18)
**Gender**			
NA	0	2	0
Female	29 (21.5%)	104 (22.4%)	26 (21.5%)
Male	106 (78.5%)	361 (77.6%)	95 (78.5%)
**BMI (kg m^{-2})**			
NA	20	53	12
Mean (sd)	27.65 (5.53)	28.15 (6.59)	26.94 (6.54)
**INTERMACS Profile**			
NA	0	3	1
1	28 (20.7%)	83 (17.9%)	24 (20.0%)
2	48 (35.6%)	163 (35.1%)	42 (35.0%)
3	44 (32.6%)	157 (33.8%)	35 (29.2%)
4	13 (9.6%)	51 (11.0%)	19 (15.8%)
5	1 (0.7%)	9 (1.9%)	0 (0.0%)
6	0 (0.0%)	1 (0.2%)	0 (0.0%)
7	1 (0.7%)	0 (0.0%)	0 (0.0%)
**Listing Status**			
BTE (Bridge to eligibility)	22 (16.4%)	55 (11.8%)	14 (11.6%)
BTT (Bridge to transplant)	36 (26.9%)	126 (27.0%)	28 (23.1%)
DT (Destination Therapy)	76 (56.7%)	286 (61.2%)	79 (65.3%)
Other (N/A)	1	0	0
**Right Atrial Pressure (mm Hg)**			
NA	11	49	18
Mean (Sd)	11.06 (6.73)	11.78 (6.75)	11.67 (6.52)
**Right Ventricular Dysfunction (from Echocardiogram)**			
NA	10	40	10
Mild	20 (16.0%)	67 (15.7%)	20 (18.0%)
Moderate/Marked	51 (40.8%)	195 (45.7%)	53 (47.7%)
Normal	25 (20.0%)	95 (22.2%)	21 (18.9%)
Severe	29 (23.2%)	70 (16.4%)	17 (15.3%)
**Ventilator Requirement**			
NA	0	1	0
No	127 (94.1%)	435 (93.3%)	113 (93.4%)
Yes	8 (5.9%)	31 (6.7%)	8 (6.6%)
**Tricuspid Regurgitation Grade (from Echocardiogram)**			
NA	5	19	4
0	17 (13.1%)	49 (10.9%)	11 (9.4%)
1+	22 (16.9%)	64 (14.3%)	17 (14.5%)
2+	46 (35.4%)	163 (36.4%)	46 (39.3%)
3+	37 (28.5%)	142 (31.7%)	36 (30.8%)
4+	8 (6.2%)	30 (6.7%)	7 (6.0%)
**Cardiac Index (L min^{-1} m^2)**			
NA	8	40	17
Mean (Sd)	2.10 (0.64)	1.97 (0.58)	1.94 (0.61)
**Heart Rate (bpm)**			
NA	1	22	8
Mean (Sd)	87.78 (17.93)	88.80 (18.99)	86.81 (20.66)
**Mean Pulmonary Artery Pressure (mm Hg)**			
NA	13	58	19
Mean (Sd)	34.89 (10.61)	36.26 (10.92)	34.50 (10.96)
**Serum Creatinine (mg dl^{-1})**			
NA	0	2	0
Mean (Sd)	1.49 (0.74)	1.51 (0.93)	1.39 (0.92)
**History of prior Cardiac Surgery**			
NA	12	41	13
No	101 (82.1%)	325 (76.3%)	80 (74.1%)
Yes	22 (17.9%)	101 (23.7%)	28 (25.9%)

**Systolic Blood Pressure (mm Hg)**			
NA	38	129	32
Mean (Sd)	93.85 (29.37)	95.01 (24.99)	96.25 (27.09)
**ALT (IU)**			
NA	0	8	0
Mean (Sd)	89.53 (297.95)	66.38 (193.55)	80.10 (173.06)
**Right Ventricular Stroke Work Index (mmHg liter m^{-2})**			
NA	18	74	26
Mean (Sd)	0.77 (0.33)	0.75 (0.36)	0.75 (0.45)

Supplementary Table 3: Baseline demographics and pre-operative clinical and hemodynamic variables for the training, validation, and testing datasets.

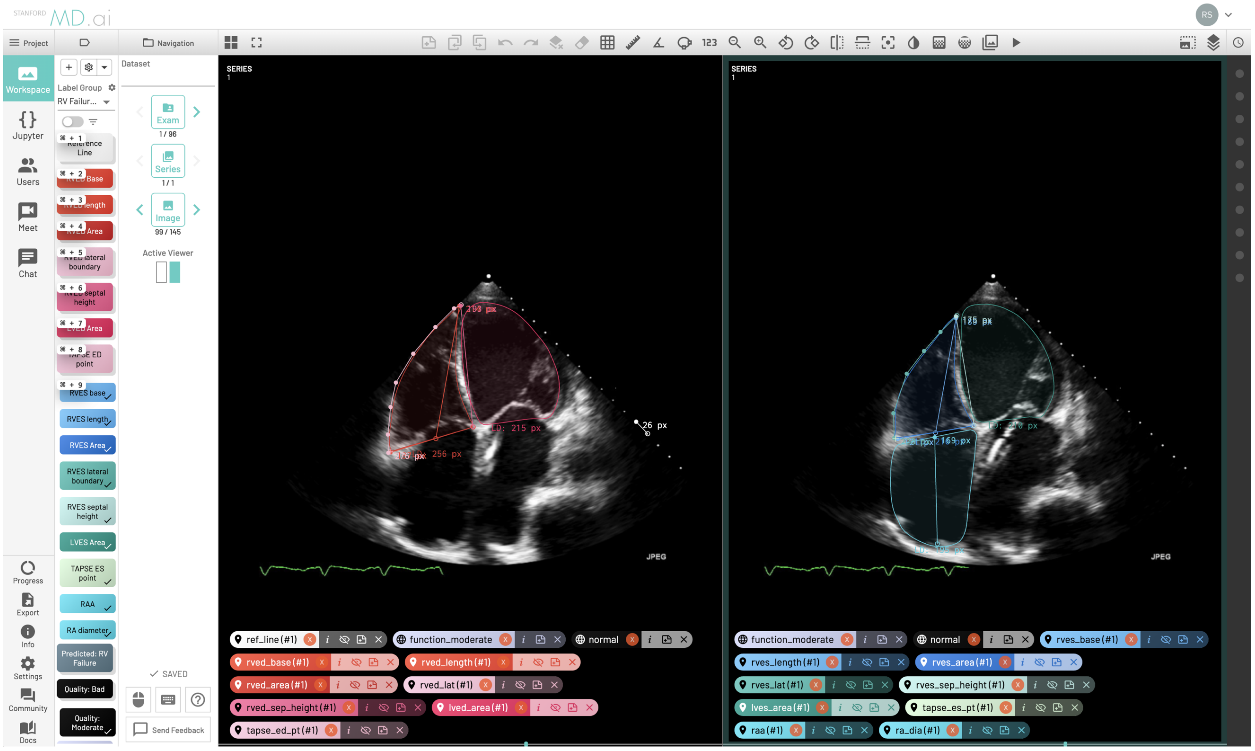

Supplementary Fig 1: Screenshot of the md.ai annotation tool with a secure Google Cloud Bucket for data archiving. Detailed measurements of ventricular and atrial metrics are made by a team of sonographers and heart failure specialists. Annotations are made in the end-diastolic and end-systolic phases, in the form of lines or polygons where appropriate. Annotation data are exported as a json file, from which vertex coordinates are mapped to a reference mapping scale. These base measurements (in cm) are then used to calculate the different clinical echocardiography metrics. Reference scripts for data annotation are available at: https://github.com/rohanshad/postop_rv_failure_echo

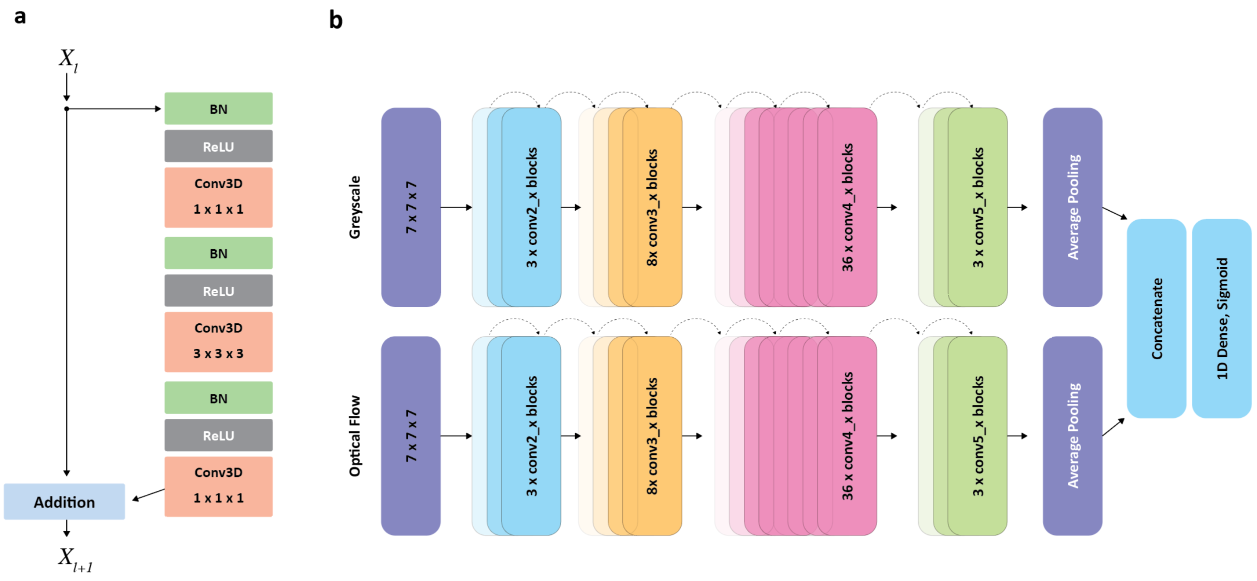

Supplementary Fig 2a. The Residual Network blocks allow for skip connections between different layers.[40] The bottleneck residual units contain in sequence: Batch Normalization, ReLU Activation, Conv3D, Batch Normalization, ReLU Activation, and then a final Conv3D layer. The output is combined with a skip connection via an Addition Layer. Each 3D Convolutional layer was additionally set with channel groups = 1, and dilation rate set to 1. **b.** Overall architecture of our two-stream network with parallel greyscale and optical flow 152-layer residual networks, converging to a single concatenation layer past each terminal average pooling layer.

Layer name	Output size	18-layer	34-layer	50-layer	152-layer
conv1	$\mathbf{112 \times 112}$	$7 \times 7 \times 7$, 64, stride 2			
conv2_x	$\mathbf{56 \times 56}$	$3\times 3 \times 3$ max pool, stride 2			
		$\begin{bmatrix} 3\times3\times3, & 64 \\ 3\times3\times3, & 64 \end{bmatrix} \times 2$	$\begin{bmatrix} 3\times3\times3, & 64 \\ 3\times3\times3, & 64 \end{bmatrix} \times 3$	$\begin{bmatrix} 1\times1\times1, & 64 \\ 3\times3\times3, & 64 \\ 1\times1\times1, & 256 \end{bmatrix} \times 3$	$\begin{bmatrix} 1\times1\times1, & 64 \\ 3\times3\times3, & 64 \\ 1\times1\times1, & 256 \end{bmatrix} \times 3$
conv3_x	$\mathbf{28 \times 28}$	$\begin{bmatrix} 3\times3\times3, & 128 \\ 3\times3\times3, & 128 \end{bmatrix} \times 2$	$\begin{bmatrix} 3\times3\times3, & 128 \\ 3\times3\times3, & 128 \end{bmatrix} \times 4$	$\begin{bmatrix} 1\times1\times1, & 128 \\ 3\times3\times3, & 128 \\ 1\times1\times1, & 512 \end{bmatrix} \times 4$	$\begin{bmatrix} 1\times1\times1, & 128 \\ 3\times3\times3, & 128 \\ 1\times1\times1, & 512 \end{bmatrix} \times 8$
conv4_x	$\mathbf{14 \times 14}$	$\begin{bmatrix} 3\times3\times3, & 256 \\ 3\times3\times3, & 256 \end{bmatrix} \times 2$	$\begin{bmatrix} 3\times3\times3, & 256 \\ 3\times3\times3, & 256 \end{bmatrix} \times 6$	$\begin{bmatrix} 1\times1\times1, & 256 \\ 3\times3\times3, & 256 \\ 1\times1\times1, & 1024 \end{bmatrix} \times 6$	$\begin{bmatrix} 1\times1\times1, & 256 \\ 3\times3\times3, & 256 \\ 1\times1\times1, & 1024 \end{bmatrix} \times 36$
conv5_x	$\mathbf{7 \times 7}$	$\begin{bmatrix} 3\times3\times3, & 512 \\ 3\times3\times3, & 512 \end{bmatrix} \times 2$	$\begin{bmatrix} 3\times3\times3, & 512 \\ 3\times3\times3, & 512 \end{bmatrix} \times 3$	$\begin{bmatrix} 1\times1\times1, & 512 \\ 3\times3\times3, & 512 \\ 1\times1\times1, & 2048 \end{bmatrix} \times 3$	$\begin{bmatrix} 1\times1\times1, & 512 \\ 3\times3\times3, & 512 \\ 1\times1\times1, & 2048 \end{bmatrix} \times 3$
	$\mathbf{1 \times 1}$	Average Pool 3D, 1D Dense, Sigmoid			

Supplementary Table 4. The initial convolutional layer has a 7 x 7 x 7 receptive field with a kernel size of 64 and stride of 2. L2 regularization of 1e-4 is used for each residual block. The different 3D ResNet depths contain varying numbers of convolutional layers for sizes 56 x 56 down to 7 x 7. The smaller 18 and 34-layer ResNets employ residual blocks without 1 x 1 x 1 bottlenecks.

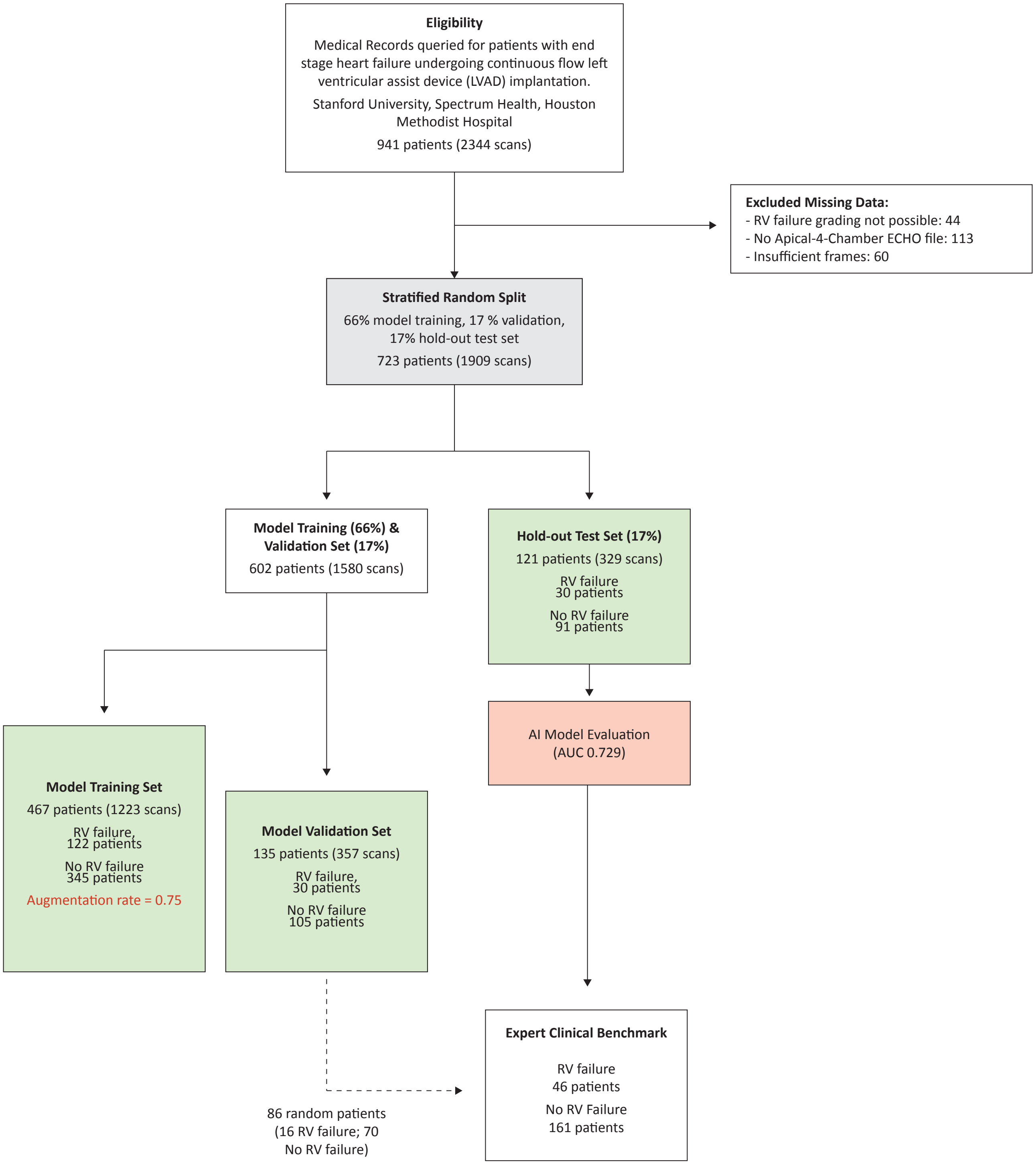

Supplementary Fig. 3: Patient records and echocardiography scans from three high volume LVAD centers in the United States were screened for eligibility. All exclusions and data splits are detailed above.

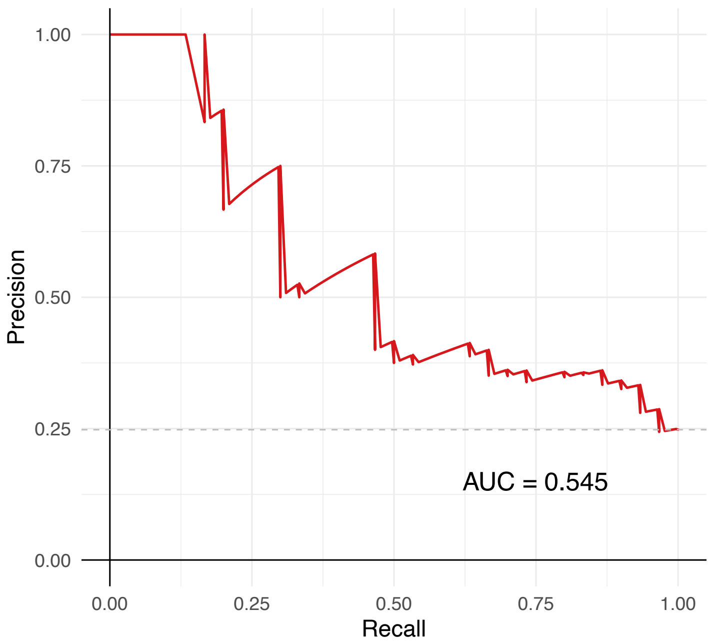

Supplementary Fig. 4: Precision-Recall Curve for the ML system. Grey dotted line denotes baseline performance

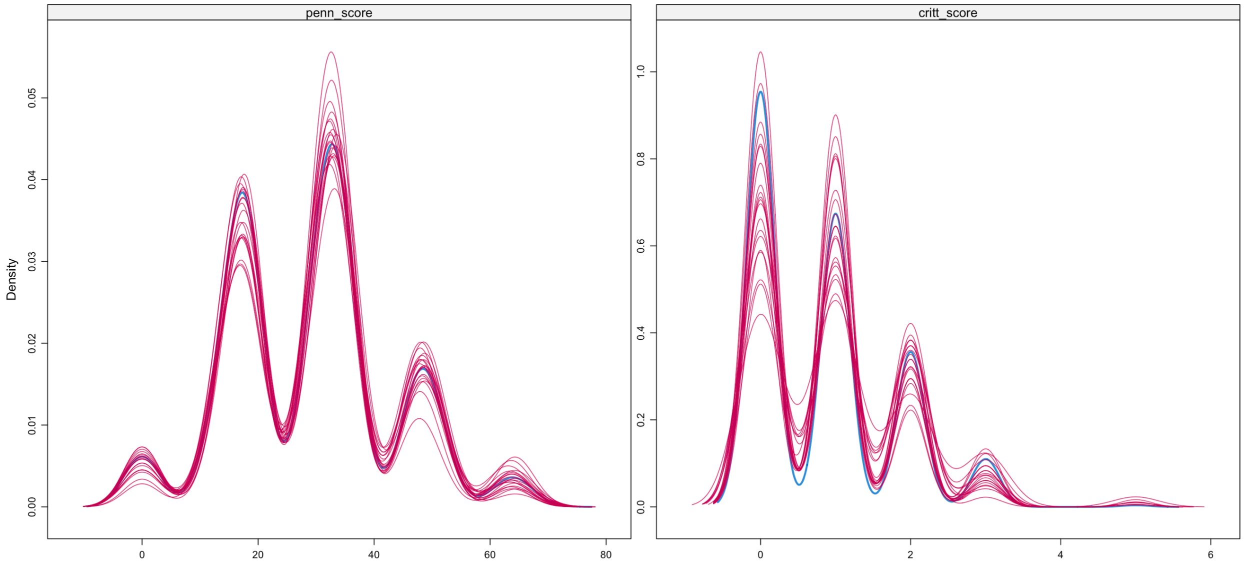

Supplementary Fig. 5: Density plots for imputed Penn score and CRITT score. Red curves indicate distribution of imputed datasets, Blue curve is the distribution of observed (non-imputed) dataset.

	Included (N=723)	Discarded (N=174)	p-value
**RV Failure Status**			0.060
No	541 (74.8%)	118 (67.8%)	
Yes	182 (25.2%)	56 (32.2%)	
**Age (years)**			0.439
Mean (SD)	57.46 (13.13)	56.59 (12.95)	
**Gender**			0.268
NA	2	9	
Female	159 (22.1%)	43 (26.1%)	
Male	562 (77.9%)	122 (73.9%)	
**BMI (kg/m^2)**			0.060
NA	85	34	
Mean (SD)	27.85 (6.41)	28.98 (6.44)	
**INTERMACS Profile**			0.015
NA	4	15	
1	135 (18.8%)	44 (27.7%)	
2	253 (35.2%)	48 (30.2%)	
3	236 (32.8%)	37 (23.3%)	
4	83 (11.5%)	28 (17.6%)	
5	10 (1.4%)	1 (0.6%)	
6	1 (0.1%)	0 (0.0%)	
7	1 (0.1%)	1 (0.6%)	
**Listing Status**			0.025
BTE (Bridge to eligibility)	91 (12.6%)	17 (10.2%)	
BTT (Bridge to transplant)	190 (26.3%)	43 (25.9%)	
DT (Destination Therapy)	441 (61.1%)	104 (62.7%)	
Other (N/A)	1	10	
**Right Atrial Pressure (mmHg)**			**< 0.001**
NA	78	37	
Mean (SD)	11.63 (6.70)	14.15 (7.15)	
**Right Ventricular Dysfunction (from echocardiogram)**			0.336
NA	60	30	
Mild	107 (16.1%)	28 (19.4%)	
Moderate/Marked	299 (45.1%)	54 (37.5%)	
Normal	141 (21.3%)	37 (25.7%)	
Severe	116 (17.5%)	25 (17.4%)	
**Ventilator Requirement**			**< 0.001**
NA	1	10	
No	675 (93.5%)	139 (84.8%)	
Yes	47 (6.5%)	25 (15.2%)	
**Tricuspid Regurgitation Grade (from echocardiogram)**			0.050
NA	28	23	
0	77 (11.1%)	24 (15.9%)	
1+	103 (14.8%)	28 (18.5%)	
2+	255 (36.7%)	45 (29.8%)	
3+	215 (30.9%)	38 (25.2%)	
4+	45 (6.5%)	16 (10.6%)	
**Cardiac Index ($L\ min^{-1}\ m^2$)**			0.418
NA	65	33	
Mean (SD)	1.99 (0.60)	2.04 (0.58)	
**Heart Rate (bpm)**			0.642
NA	31	27	
Mean (SD)	88.27 (19.06)	87.48 (18.19)	
**Mean Pulmonary Artery Pressure (mm Hg)**			0.010
NA	90	40	
Mean (SD)	35.71 (10.87)	38.33 (9.73)	
**Serum Creatinine ($mg\ dl^{-1}$)**			0.591
NA	2	11	
Mean (SD)	1.49 (0.89)	1.53 (0.95)	
**History of prior cardiac surgery**			0.174
NA	66	89	
No	506 (77.0%)	71 (83.5%)	
Yes	151 (23.0%)	14 (16.5%)	

**Systolic Blood Pressure (mm Hg)**			**< 0.001**
NA	199	119	
Mean (SD)	95.00 (26.17)	82.36 (31.59)	
**ALT (IU)**			0.007
NA	8	13	
Mean (SD)	73.07 (214.04)	134.73 (408.10)	
**Right Ventricular Stroke Work Index (mmHg liter m^{-2})**			0.051
NA	118	46	
Mean (SD)	0.75 (0.37)	0.82 (0.45)	

Supplementary Table 5: Baseline characteristics and demographics of patients who had sufficient information for determination of RV failure status (n=897). 173 patient records were discarded due to insufficient frames in echocardiography videos (60), and no available apical 4 chamber scan (113). Two-sided students t-tests were used to compare continuous variables, Chi-squared tests were used to compare categorical variables. No adjustments were made for multiple comparisons.

	No RV Failure (N=161)	RV Failure (N=46)	p value
**RV End diastolic base (cm)**			0.092
Mean (SD)	4.10 (1.68)	4.56 (1.53)	
**RV End diastolic length (cm)**			0.447
Mean (SD)	7.78 (2.98)	8.15 (2.51)	
**RV End diastolic area (cm^2)**			0.122
Mean (SD)	22.70 (13.88)	26.27 (13.10)	
**RV End diastolic septal height (cm)**			0.379
Mean (SD)	7.23 (2.74)	7.62 (2.34)	
**Tricuspid Annular Plane Excursion (TAPSE) (cm)**			0.926
Mean (SD)	1.15 (0.99)	1.13 (0.63)	
**RV Ejection Fraction (RVEF) (%)**			0.771
Mean (SD)	20.30 (10.86)	19.74 (13.52)	
**RV Longitudinal Strain (RVLS) (%)**			0.389
Mean (SD)	-25.97 (6.78)	-27.00 (8.06)	
**Right Atrial Area (cm^2)**			0.301
Mean (SD)	17.59 (11.16)	19.45 (9.09)	
**Right Atrial Diameter (cm)**			0.114
Mean (SD)	4.41 (1.77)	4.86 (1.48)	
**LV End Systolic Area (cm^2)**			0.422
Mean (SD)	37.96 (23.14)	40.97 (19.30)	
**LV End Diastolic Area (cm^2)**			0.380
Mean (SD)	42.67 (25.40)	46.29 (21.03)	
**RV End Systolic Area (cm^2)**			0.117
Mean (SD)	18.17 (11.54)	21.23 (11.79)	
**Qualitative Clinical Assessment**			0.075
Severe dysfunction	55 (34.16%)	23 (50.0%)	
Normal / Milder dysfunction	106 (65.84%)	23 (50.0%)	

Supplementary Table 6: List of echocardiographic measurements taken as part of the clinical expert evaluation, including additional qualitative clinical assessments of cardiac function. Area measurements were made in the appropriate phase of the cardiac cycle via manual segmentation in the apical 4-chamber view. TAPSE It is measured as the length between the end-diastolic and peak systolic points of the lateral tricuspid annulus. RV longitudinal strain was calculated from mid-endocardial end-diastolic and end-systolic manually traced lengths of the RV free wall, and expressed as absolute values, as previously described. Two-sided students t-tests were used to compare continuous variables, Chi-squared tests were used to compare categorical variables. No adjustments were made for multiple comparisons.

Model Architectures	AUC (95% CI)	Kinetics 600 pretrained
Resnet-18	0.533 (0.456 – 0.609)	No
Resnet-50	0.604 (0.319 – 0.890)	No
Resnet-152	0.556 (0.438 – 0.676)	No
Resnet-18	0.631 (0.554 – 0.709)	Yes
Resnet-50	0.702 (0.592 – 0.811)	Yes
Resnet-152	0.697 (0.589 – 0.804)	Yes
Resnet-18 + EHR	0.644 (0.525 – 0.762)	Yes
Resnet-50 + EHR	0.682 (0.617 – 0.746)	Yes
Resnet-152 + EHR	0.739 (0.642 – 0.837)	Yes
Resnet-18 + 224px input	0.673 (0.601 – 0.746)	Yes
Resnet-18 + 224px input	0.599 (0.522 – 0.675)	No
Resnet-50 + optical flow	0.686 (0.619 – 0.752)	Yes
Resnet-152 + optical flow	0.749 (0.634 – 0.863)	Yes
TimeSformer		Yes

Supplementary Table 7: Validation set performance of various architectures, pretraining on Kinetics-600 video action recognition dataset, and input streams (pure video, video + EHR, video + optical flow).

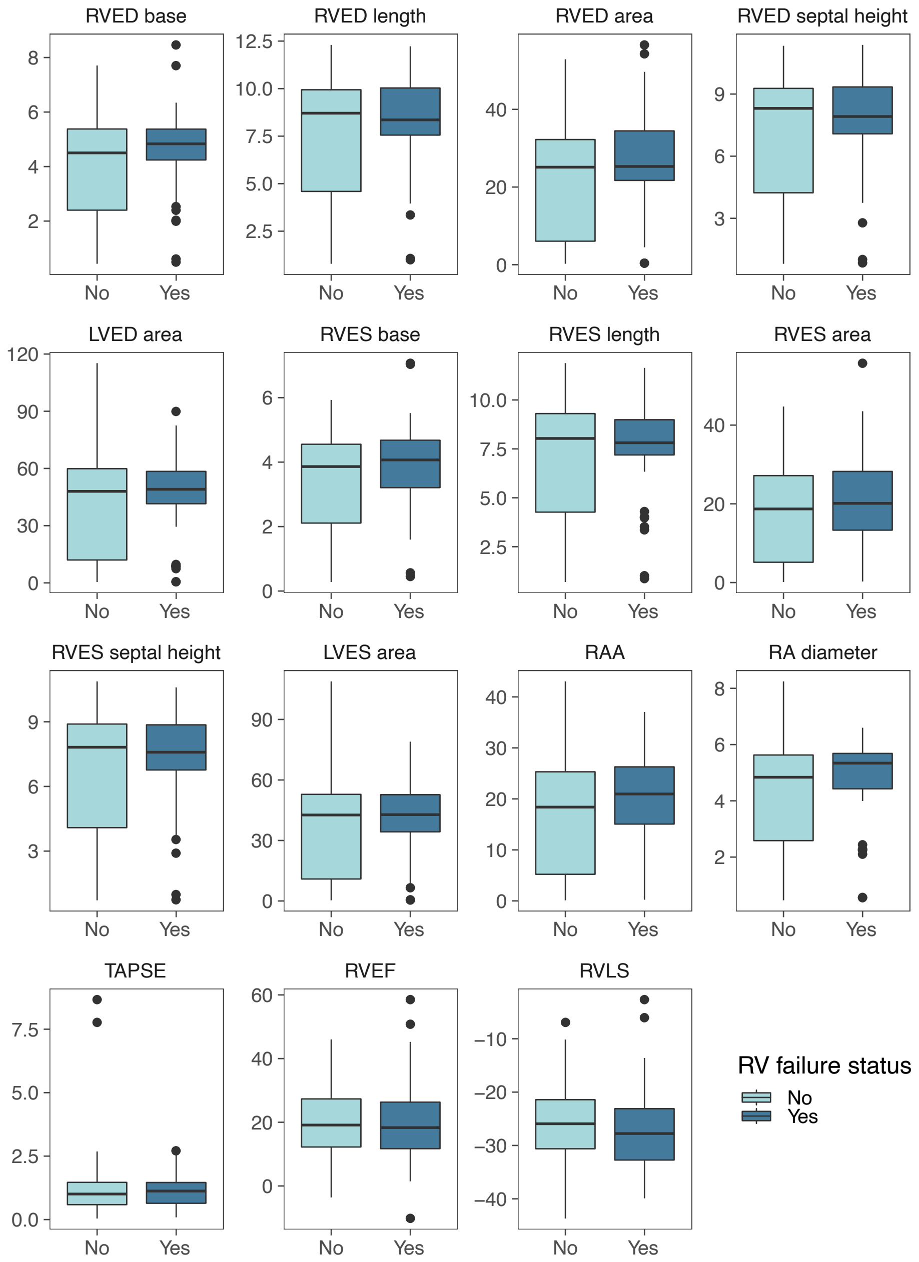

Supplementary Fig. 6: Boxplots stratified by RV failure status for expert clinician measurements in the clinical benchmark study (n = 207; 161 with RV failure and 46 without). RVED base, RVED length, RVED septal height, TAPSE, RA diameter (cm); RVED area, RA area, LVES area, LVED area, RVES area (cm^2); RVEF and RVLS (%). Center line of the boxplots denotes median and bounds are the 25th and 75th percentile. Whiskers terminate at 1.5 IQR from the bounds of the boxplots.
