# Supplementary material for: Predicting post-operative right ventricular failure using video-based deep learning
Source: Nat Commun. 2021 Aug 31;12:5192. doi: 10.1038/s41467-021-25503-9 (PMC8408163; doi:10.1038/s41467-021-25503-9)
Supplement: Supplementary file 2 — Reporting Summary [file 41467_2021_25503_MOESM2_ESM.pdf]

nature research

Corresponding author(s): William Hiesinger

Last updated by author(s): 8/1/2021

Reporting Summary

Nature Research wishes to improve the reproducibility of the work that we publish. This form provides structure for consistency and transparency in reporting. For further information on Nature Research policies, see our Editorial Policies and the Editorial Policy Checklist.

Statistics

For all statistical analyses, confirm that the following items are present in the figure legend, table legend, main text, or Methods section.

n/a	Confirmed	
☐	☒	The exact sample size (n) for each experimental group/condition, given as a discrete number and unit of measurement
☐	☒	A statement on whether measurements were taken from distinct samples or whether the same sample was measured repeatedly
☐	☒	The statistical test(s) used AND whether they are one- or two-sided<br>*Only common tests should be described solely by name; describe more complex techniques in the Methods section.*
☒	☐	A description of all covariates tested
☒	☐	A description of any assumptions or corrections, such as tests of normality and adjustment for multiple comparisons
☐	☒	A full description of the statistical parameters including central tendency (e.g. means) or other basic estimates (e.g. regression coefficient) AND variation (e.g. standard deviation) or associated estimates of uncertainty (e.g. confidence intervals)
☐	☒	For null hypothesis testing, the test statistic (e.g. F, t, r) with confidence intervals, effect sizes, degrees of freedom and P value noted<br>*Give P values as exact values whenever suitable.*
☒	☐	For Bayesian analysis, information on the choice of priors and Markov chain Monte Carlo settings
☒	☐	For hierarchical and complex designs, identification of the appropriate level for tests and full reporting of outcomes
☒	☐	Estimates of effect sizes (e.g. Cohen's d, Pearson's r), indicating how they were calculated

Our web collection on statistics for biologists contains articles on many of the points above.

Software and code

Policy information about availability of computer code

Data collection	Code for this study will be made available at https://github.com/rohanshad/postop_rv_failure_echo
Data analysis	Data analysis was performed using R 3.6.2 and python 3.7.7. We used the TensorFlow (2.1.0) deep learning library, numpy (1.18.1), opencv-python (4.5.1), scikit-learn (0.22.2). All relevant dependencies are listed in the requirements.txt files located in the code repository. Statistical methods utilized the pROC package (1.17) and dplyr (1.0.7) in R, which are detailed in the methods section of the manuscript.

For manuscripts utilizing custom algorithms or software that are central to the research but not yet described in published literature, software must be made available to editors and reviewers. We strongly encourage code deposition in a community repository (e.g. GitHub). See the Nature Research guidelines for submitting code & software for further information.

Data

Policy information about availability of data

All manuscripts must include a data availability statement. This statement should provide the following information, where applicable:

- Accession codes, unique identifiers, or web links for publicly available datasets
- A list of figures that have associated raw data
- A description of any restrictions on data availability

Restrictions apply to availability of the multi-center data used for training and evaluating our models, due to the nature of the governing data use and transfer agreements. The dataset for this study was acquired under data transfer agreements that restrict public release due to the potential embedded protected health information present in the raw data. Access to subsets of the data can be obtained as follows: Please contact Guha A (gashrith@houstonmethodist.org). and Lee S (Sangjin.Lee@spectrumhealth.org). for access to paired raw imaging and patient level data from Houston Methodist and Spectrum Health respectively. Access may be granted for research use subject to institutional ethical approvals. Please contact Hiesinger W (willhies@stanford.edu). for access to raw imaging data and patient level data from the Stanford cohort. Access to all Stanford subsets (training / validation / testing) will be granted to all accredited researchers pending

approvals of relevant data use agreements via Stanford's office of sponsored research. The EchoNet Dynamic dataset is publicly available at: https://echonet.github.io/dynamic/. The Kinetics action recognition dataset is publicly available at: https://deepmind.com/research/open-source/kinetics.

Field-specific reporting

Please select the one below that is the best fit for your research. If you are not sure, read the appropriate sections before making your selection.

☒ Life sciences ☐ Behavioural & social sciences ☐ Ecological, evolutionary & environmental sciences

For a reference copy of the document with all sections, see nature.com/documents/nr-reporting-summary-flat.pdf

Life sciences study design

All studies must disclose on these points even when the disclosure is negative.

Sample size	No sample size calculations were performed a-priori. Deep learning models were trained while varying the number of input videos until asymptotic improvement of model performance was achieved to suggest an appropriate sample size was obtained. This dataset size proved sufficient for training the models and evaluating their performance. However the performance of the models would likely improve with increased sample size.
Data exclusions	Prior to analyses, forty four records were discarded due to missing data on duration of post-operative inotropes that prevented the adjudication of RV failure status. Data from an additional 173 patients were discarded because of missing apical 4-chamber echocardiograms or insufficient number of frames per video (Supplementary Fig. 3 and Supplementary Table 5).
Replication	All attempts at reproducing the results (ensemble of 3 independently trained models) were successful on the hold-out test dataset. No other attempts at replication were made.
Randomization	The training, validation, and test datasets split to approximately (66%/17%/17%) was performed using a random stratified sampling method to ensure each split had approximately the same proportion of patients with and without RV failure.
Blinding	The clinical team was blinded to the outcomes of each patients in our clinical benchmarking study

Reporting for specific materials, systems and methods

We require information from authors about some types of materials, experimental systems and methods used in many studies. Here, indicate whether each material, system or method listed is relevant to your study. If you are not sure if a list item applies to your research, read the appropriate section before selecting a response.

Materials & experimental systems

n/a	Involved in the study	
☒	☐	Antibodies
☒	☐	Eukaryotic cell lines
☒	☐	Palaeontology and archaeology
☒	☐	Animals and other organisms
☐	☒	Human research participants
☒	☐	Clinical data
☒	☐	Dual use research of concern

Methods

n/a	Involved in the study	
☒	☐	ChIP-seq
☒	☐	Flow cytometry
☒	☐	MRI-based neuroimaging

Human research participants

Policy information about studies involving human research participants

Population characteristics	All patients aged 18-years or older with at least one pre-operative transthoracic echocardiogram as well as a complete pre-operative and post-operative assessment of RV failure during index-hospitalization as per the MCS-ARC consensus definitions. The dataset consisted of 159 (21.9%) females, and 562 (77.6%) male patients with an average age of 57.4 (sd 13.1) years. Additional baseline characteristics and demographics by data split are outlined in Supplementary Table 3.
Recruitment	Data were retrospectively sourced from the departments of Cardiothoracic Surgery at Stanford University (CA), Spectrum Health Grand Rapids (MI), and the Houston Methodist Hospital (TX). As described in the manuscript, all patients aged 18-years or older with at least one pre-operative transthoracic echocardiogram as well as a complete pre-operative and post-operative assessment of RV failure during index-hospitalization as per the MCS-ARC consensus definitions (Mechanical circulatory assist academic research consortium) were included (Supplementary Table 1). As a result, we do not anticipate self selection biases. The results are however biased to reflect practice in the setting of high volume clinical centers.
Ethics oversight	Stanford University Institutional Review Board (IRB 52440)

Note that full information on the approval of the study protocol must also be provided in the manuscript.
